# Psychological Screening and Support for Patients Enrolled in Early‐Phase Cancer Clinical Trials and the Possible Barriers: A Real‐World Tertiary Center Implementation Study

**DOI:** 10.1002/pon.70446

**Published:** 2026-04-06

**Authors:** Matthew D. Robinson, Ruiyang Yan, Amy Smith, Harriet Morley, Louise Carter, Donna Graham, Matt Krebs, Fiona Thistlethwaite, Natalie Cook

**Affiliations:** ^1^ Experimental Cancer Medicine Team The Christie Hospital NHS Foundation Trust Manchester UK; ^2^ Queen's Hospital Barking, Havering and Redbridge University Hospitals London UK; ^3^ Division of Cancer Sciences The University of Manchester Manchester UK

## Abstract

**Introduction:**

One in three patients with cancer suffers from clinically significant depression and/or anxiety. Early‐phase cancer clinical trials (EPCCTs) frequently include patients with advanced disease with limited treatment options. However, screening for psychological symptoms is not routinely performed in this setting. Here, we describe a mixed‐methods implementation study exploring the feasibility of improving access to psychological screening in EPCCTs and assessing how electronic patient‐reported outcome measures (ePROMs) can be utilized to identify those requiring intervention.

**Methods:**

Perceived benefits and challenges associated with psychological screening in EPCCTs were explored from the clinician, psycho‐oncology team, and patient and carer's perspectives, via clinician surveys and a patient and carer focus group. A standard workflow was created to enable routine psychological screening, alongside a stepped‐care model for providing support. An ePROM, consisting of PHQ‐2 and GAD‐7 questionnaires, was distributed to patients enrolling on EPCCTs.

**Results:**

Of the 17 clinicians who responded, 88% felt that routine psychological assessments were important, and 100% were willing to complete assessments with patients. Perceived barriers were identified relating to 4 main themes: resources, support services, training and staff misconceptions. Invitations to ePROMs were sent to 92 patients over 12 months and 59 (64.1%) have responded at baseline; of these, 13 (22.0%) patients have received intervention based on PHQ‐2/GAD‐7 score. Initial challenges associated with ePROM completion related to technological difficulties, missed notifications and form expiration.

**Conclusion:**

Our findings suggest that routine psychological assessments in EPCCTs may help identify previously undiagnosed psychological burden in patients, and facilitate their access to appropriate, timely support. This implementation study indicates this approach can be feasibly delivered through the use of ePROMs.

## 1 Introduction

One in three patients diagnosed with cancer suffers from clinically significant anxiety or depression, and, in one study were over five times more likely to develop depression than the general population [1]. Factors associated with increased prevalence of psychological distress include disease type, stage of cancer and treatment setting [1, 2, 3]. Early phase cancer clinical trials (EPCCTs) include phase I and II studies that aim to investigate dosing, interrogate safety and tolerability and examine the effectiveness of a new therapeutic agent or combination of agents. These studies frequently include patients with advanced disease, poor prognosis and limited treatment options, which correlate with an increased psychological burden [4]. This in turn is associated with increased morbidity and mortality [5]. The literature advocates for the introduction of regular psychological screening in patients diagnosed with cancer, not only to alleviate psychological suffering but to mitigate the associated sequelae, from reduced quality of life and treatment adherence to increased risk of suicide and exacerbation of somatic symptoms [6]. The American Society of Clinical Oncology recommends that all patients diagnosed with cancer or cancer survivors are evaluated for symptoms of anxiety and depression periodically throughout the course of their disease [7], whilst the European Society for Medical Oncology advises that the generalized anxiety disorder questionnaire (GAD‐7) and hospital anxiety and depression scale (HADS) are appropriate tools to screen for anxiety, along with Beck's depressive inventory, and the patient health questionnaire (PHQ‐9) for depression [8].

Less than 30% of oncology clinical trials registered between 2007–2013 incorporated patient‐reported outcome measures into their trial protocols [9]. Given that clinical trial outcome measures frequently omit metrics relating to psychological well‐being, symptoms of anxiety and depression in this population may remain undetected, identifying a significant area of unmet need.

The psychological impact of EPCCTs on patients has previously been investigated [10]. From 57 patients entering an EPCCT at the Christie Hospital, anxiety and depression were reported in 39% and 18% of patients, respectively. Notably, 63% of participants experiencing psychological distress had not previously reported this, highlighting a significant need for psychological screening in this clinical setting to identify these patients and provide appropriate support.

Alongside this, if patients are discovered to have clinically relevant levels of psychological distress through screening, a pathway must be in place to guide patients toward appropriate interventions. The stepped care model has been utilized in a wide range of healthcare settings, which aims to provide the most clinically effective treatment in the least resource‐dependent way possible. This approach to care delivery facilitates variety in the type of intervention, the duration of treatment and the method of delivery, enabling a degree of personalized care that is complimentary to the patient's preferences and the available resources [11, 12, 13].

Patient‐reported outcome measures enable the collection of a wide range of health‐related data from patients, often including symptom burden, treatment‐related adverse effects and quality of life, without interpretation of the patient's response by a clinician or anyone else. The use of electronic patient‐reported outcome measures (ePROMs) in routine cancer care and palliative inpatient settings has been linked to improved symptom control, enhanced patient‐clinician communication, and improved survival rates [12, 13, 14]. However, their utility within EPCCTs remains relatively unexplored.

Here, we describe a service evaluation of an ePROM‐based psychological screening tool implemented for patients entering EPCCTs at a specialist cancer hospital, to assess acceptability, workflow integration, and implementation barriers rather than clinical effectiveness.

## 2 Materials and Methods

### 2.1 Identifying and Assessing Barriers

A healthcare professional survey including 10 open and closed questions (Supporting Information S1: Figure S1) was distributed to all clinical members of the multidisciplinary team based in the experimental cancer medicine team (ECMT) of The Christie NHS Foundation Trust, a specialist cancer hospital in the UK. The ECMT specializes in treating patients with multiple cancer types enrolled in phase I/II clinical trials (EPCCTs).

Patients enrolled in a clinical trial within the ECMT, along with their family members, were invited to participate in a patient and carer focus group. The number of participants in the focus groups was guided by the need for data adequacy and depth of discussion, with a target of five to seven participants to allow meaningful interaction while maintaining a comfortable setting for discussing sensitive psychological issues. For the staff survey, all multidisciplinary team members actively involved in EPCCT delivery were invited to participate. The focus groups were facilitated by a senior research nurse and senior clinical trials coordinator. Eleven open‐ended, predefined questions were used to guide the discussion (Supporting Information S1: Figure S2), with flexibility to explore emerging ideas. Discussions were recorded, transcribed verbatim, and reviewed alongside audio recordings to ensure accuracy.

Qualitative data from open‐ended staff survey responses and focus group transcripts were analyzed using an inductive thematic analysis approach, following the framework described by Braun and Clarke. Transcripts were reviewed in detail by HM to allow familiarization with the data and generation of initial codes. Interpretation was supported by contemporaneous notes taken by the senior research nurse during the focus groups, which were used to verify transcript accuracy and inform theme development. Themes and sub‐themes were developed iteratively with reference to the underlying data. Given the feasibility‐ and implementation‐focused nature of this service evaluation, data adequacy rather than formal thematic saturation was prioritized.

A virtual meeting was held with the local psycho‐oncology team to discuss barriers from their perspective, using a semi‐structured interview format. This meeting included a senior clinical trials coordinator, a medical oncology consultant, a research project manager, 4 nurse specialists, a student nurse, a consultant psychiatrist, a counselor/psychotherapist, and a specialty doctor.

### 2.2 Electronic Patient‐Reported Outcome Measures

MyChristie‐MyHealth is a software package launched by The Christie NHS Foundation Trust in 2019 that facilitates the integration of ePROM questionnaires into patient care pathways [15]. An ePROM questionnaire, based on PHQ‐2 and GAD‐7, was developed to screen for symptoms of anxiety and depression (Supporting Information S1: Figure S3). All patients enrolled on an EPCCT between March 2024 and March 2025 were approached and invited to participate consecutively, without formal power calculation, as the primary aim was feasibility and service development. Those who agreed to complete it received an access link via email or SMS. Individual responses were automatically uploaded onto the local electronic patient record at The Christie and then reviewed by a clinician.

### 2.3 Stepped Care Model

**FIGURE 1:**
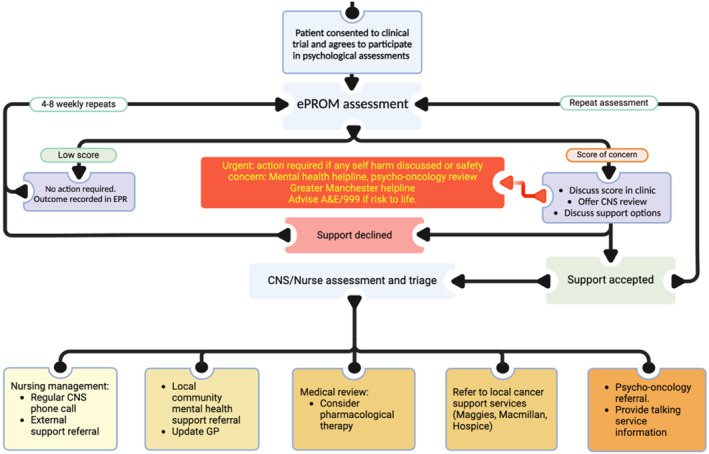
A schematic of the psychological assessment workflow and stepped care model of support. CNS, Clinical Nurse Specialist; EPR, electronic patient record; ePROM, electronic patient report outcome measure; GP, general practitioner.

To streamline management options appropriately, a stepped‐care model of support was devised based on the scores generated by the ePROM. Patients could view their scores immediately after completing the questionnaire. For those with a low score of no concern (PHQ‐2 ≤ 2, GAD‐7 ≤ 4), no intervention was required (Figure 1); however, they were encouraged to inform clinical staff if they experienced any changes in their mental health or well‐being.

The PHQ‐2 and GAD‐7 were selected due to their brevity, validation, and suitability for routine screening within time‐limited EPCCT workflows. For depressive symptoms, a PHQ‐2 score of ≥ 3 was used to indicate the need for further assessment [16, 17]. For anxiety symptoms, a GAD‐7 score of ≥ 5 was used, corresponding to at least mild anxiety based on established scoring guidelines [18]. This lower threshold was chosen to maximize sensitivity [19]. Patients scoring above these thresholds were flagged for clinical review and discussion within the stepped‐care framework.

Depending on their preference, patients could accept or decline further support. The additional support options included nurse‐led management with regular phone contact, referral to local mental health services with the involvement of their general practitioner (GP), initiation of appropriate pharmacological therapies, or referral to local cancer support organizations or the psycho‐oncology team. In cases where urgent intervention was necessary at any point, patients were signposted or referred directly to an urgent mental health helpline, the psycho‐oncology urgent review team, or relevant emergency services.

### 2.4 Evaluating Patient Experience

Patients who had been invited to complete the psychological assessment ePROM were contacted by phone and invited to complete a Patient Reported Experience Measure (PREM) questionnaire verbally. The PREM questionnaire was developed as an adaptation of one designed by the MyChristie‐MyHealth team previously [20].

The questionnaire contained five questions, four of which utilized a Likert scale with 4 possible answers (Supporting Information S1: Figure S4). Patients were able to answer “Not applicable” if they had not yet completed the questionnaire at the time of interviewing. Question 5 allowed free text for patients to provide feedback on any specific challenges associated with the completion of the ePROM.

### 2.5 Data Analysis

Descriptive statistics were generated for the quantitative data gathered from the staff survey, focus groups, ePROM psychological assessment questionnaire and PREM questionnaire. Inductive thematic analysis was used to analyze the qualitative data from the staff survey and the transcriptions from the focus group sessions.

### 2.6 Ethical Approval

The project was approved as a service evaluation (reference number: 4315) by The Christie NHS Foundation Trust Governance Panel. The work involved the implementation of a psychological screening pathway within routine clinical care, without randomization, experimental manipulation, or deviation from standard treatment pathways. Data were collected and analyzed for service improvement and feasibility assessment purposes rather than hypothesis‐driven research. Service evaluation in England is exempt from ethics committee review.

## 3 Results

### 3.1 Staff Survey

All 27 clinical staff members actively involved in EPCCT delivery were invited to participate in the staff survey; 17 (63%) responded, including 5 research nurses, 10 physicians, and 2 research practitioners (data not displayed). When asked if routine psychological assessments for EPCCT patients were important, 15 (88%) answered “yes”, 1 (6%) answered “no” and 1 was “unsure” (6%). All respondents felt that patients would be willing to complete psychological assessments. When asked to rate on a scale of 1–10 (1 = very easy, 10 = very difficult) how difficult they think it would be to implement psychological assessments for EPCCT patients, responses ranged from 2 to 9 (median 5.5).

Eight (47%) respondents felt that the best setting to complete psychological assessments was during a clinical appointment, whilst the waiting area and home‐based testing were favored by 4 respondents each. One individual felt that this was dependent on patient preferences. When asked how often patients should be assessed, 2 selected before treatment only, whilst 15 selected all available time points: before treatment initiation, during periods of active treatment and after treatment completion. When asked to rate on a scale of 1–10 (1 = very uncomfortable, 10 = very comfortable) how comfortable they would feel asking patients to complete psychological assessments and discussing patients' mental well‐being, responses ranged from 4 to 10 (median response was 7.5).

Results from inductive thematic analysis of the qualitative data generated by the staff survey identified six primary themes with multiple associated subthemes (Supporting Information S1: Figure S5).

### 3.2 Patient and Carer Focus Groups

A total of 5 patients and 2 of the patient's carers were recruited for the focus groups. Inclusion criteria stipulated that participants had a cancer of any type and had been screened to participate in an EPCCT.

**FIGURE 2:**
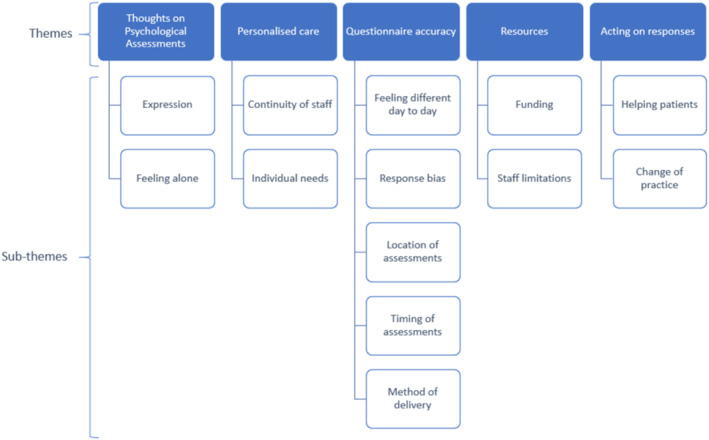
Primary themes and sub‐themes of barriers to psychological assessments derived from patient and carer focus groups.

All participants agreed that psychological assessments were important for patients participating in clinical trials. When asked to rate the importance of psychological assessments from 1 (not important) to 10 (very important), 6 (86%, 2 carers and 4 patients) answered 10, and 1 (14%, 1 patient) answered 9. Results from inductive thematic analysis of the focus group transcripts identified 5 primary themes with multiple associated subthemes (Figure 2).

### 3.3 Psycho‐Oncology Team Meeting

Barriers limiting the implementation of psychological screening were discussed with members of the psycho‐oncology team. Discussions centered around four main areas: (1) lack of resources and increasing workloads, (2) additional training requirements for staff, (3) staff misconceptions around who can provide psychological support, and (4) the importance of timely referral and collaborative attitudes toward psychological support, rather than systematic reliance on referral to psycho‐oncology.

### 3.4 Patient‐Reported Outcome Measure Questionnaire

Between March 2024 and March 2025, 94 patients enrolling on an EPCCT were approached to participate in psychological screening, of whom 92 (97.9%) consented and 2 declined participation.

Invitations to complete the baseline ePROM were sent to participants at least 3 days prior to treatment initiation. Of the 92 patients who consented, 59 (64.1%) completed the baseline ePROM questionnaire and were included in the descriptive analysis (Table 1). Among these 59 participants, slightly over half of the participants were male (52.5%), and the median age was 62, ranging from 19 to 87 years. Patients had disease originating from a wide range of primary sites, the most common being colorectal (20.3%) and lung cancer (16.9%). The majority of patients had stage IV disease (91.5%) at the time of referral to the EPCCT. No patients had a documented history of depression; 5.1% had a history of anxiety.

**TABLE 1:** Baseline patient demographics.

	Patients who completed baseline assessment EPROM (*N* = 59)
Gender, *N*, %	
Male	31 (52.5)
Female	28 (47.5)
Age, median (range)	62 (19–87)
Disease group, *N*, %	
Colorectal	12 (20.3)
Lung	10 (16.9)
Head and Neck	6 (10.2)
Bladder	5 (8.5)
Breast	4 (6.8)
Ovarian	4 (6.8)
Cervical	3 (5.1)
Prostate	3 (5.1)
Melanoma	2 (3.4)
Mesothelioma	2 (3.4)
Pancreas	2 (3.4)
Anal	1 (1.7)
Bone	1 (1.7)
Cholangiocarcinoma	1 (1.7)
Peritoneal	1 (1.7)
Renal	1 (1.7)
Thyroid	1 (1.7)
Disease stage at referral, *N*, %	
Unknown	1 (1.7)
I	0 (0.0)
II	0 (0.0)
III	4 (6.8)
IV	54 (91.5)
Known anxiety/depression, *N*, %	
No	56 (94.9)
Anxiety	3 (5.1)
Depression	0 (0)

**FIGURE 3:**
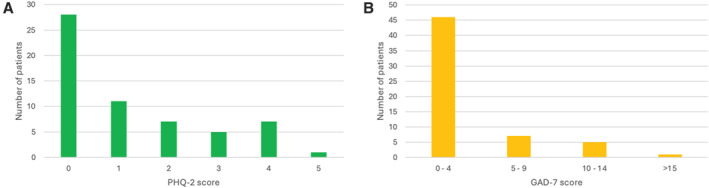
Baseline psychological assessment scores for patients enrolled on EPCCTs at The Christie. (A) PHQ‐2; (B) GAD‐7. For PHQ‐2, a score of 3 or higher suggests elevated symptoms of depression. For GAD‐7, a score of 0–4 suggests minimal anxiety, 5–9 suggests mild anxiety, 10–14 moderate anxiety, and 15 or higher suggests severe anxiety.

Median baseline PHQ‐2 and GAD‐7 scores were 0 (range, 0–4) and 2 (range, 0–17), respectively (Figure 3A, B). Thirteen (22.0%) patients had a PHQ‐2 score of ≥ 3, which suggests elevated symptoms of depression that require further assessment. For GAD‐7, 46 (78.0%) patients scored 0–4 (minimal anxiety) at baseline. Using our threshold of ≥ 5, a total of 13 (22.0%) patients scored above the cutoff for further assessment: seven (11.9%) scored between 5–9 (mild anxiety), five (8.5%) scored 10–14 (moderate anxiety), and one patient scored above 15 (severe anxiety). In all, 3 participants had a history of anxiety (Table 1). Two of these participants did not score over the minimal threshold for intervention for either PHQ2/GAD7, and one had a PHQ score of 3 (significant) and 6 (mild anxiety) for PHQ2 and GAD7, respectively.

The 19 unique patients whose scores exceeded the threshold for further assessment were reviewed by the Clinical Nurse Specialist (CNS) to assess whether additional support was needed. Six patients declined support, stating they did not want or feel they needed it; however, regular follow‐up associated with clinical trial participation continued. The remaining 13 patients received interventions, including regular CNS follow‐ups, initiation of antidepressant or anxiolytic medications, referral for complementary therapies, or referrals to hospital or community supportive oncology services.

### 3.5 Patient‐Reported Experience Measure Questionnaire

Out of 23 patients invited to participate in an interview to complete the PREM questionnaire and share their experience with ePROMs, 12 consented to take part. Among these, 9 had completed the ePROM by the time of the interview. From this, 67% of patients strongly agreed that the steps to get to the psychological assessment questionnaire were easy to follow, 89% strongly agreed that the questionnaire was easy to understand and took an acceptable amount of time to complete, and 67% strongly agreed that the questionnaire was a useful way of communicating how they are feeling (Supporting Information S1: Figure S6). All other participants agreed with questions 1–4.

No problems were experienced by 78% of patients who had completed the questionnaire. One patient had completed a paper version of the form as they felt that they were likely to make mistakes with the electronic version. Another patient reported they did not have sufficient time to complete it before the link to the electronic form expired.

Three patients had not completed the ePROM questionnaire at the time of interviewing: one reported that the link had expired and they could not access the questionnaire; one was unable to locate the email invitation and expressed a preference for SMS invitations, and the other patient misunderstood the timing.

Several logistical challenges were identified during the initial rollout of psychological assessments, and these are summarized in Table 2.

**TABLE 2:** Challenges and proposed solutions associated with the implementation of ePROM‐based psychological screening in EPCCTs.

Challenge	Proposed solution
Patients may have difficulties with completing electronic forms	Establish whether there is a preference for paper forms during the consent process. Record and communicate this preference.
Patients may agree to participate but fail to return completed forms	Contact patients with incomplete forms, as simple issues may be limiting participation.
Clinical staff may unintentionally omit psychological screening consent in their consultations	Reminder cards are now added to patient notes for clinician benefit.
There is no standard mechanism of communicating whether patients have agreed to participate	Clinicians are encouraged to document consent in the patient notes or inform the relevant CNS of patient participation.
Language barriers can make ePROM completion challenging	A paper version of the ePROM can be made available in languages other than English.
Invitation links may expire after a period of time	Patients that have not returned an ePROM form should be contacted to determine whether another invitation is needed.

## 4 Discussion

### 4.1 Summary of Descriptive Findings

This mixed‐methods implementation study assessed the feasibility and acceptability of integrating an ePROM‐based psychological screening pathway into EPCCTs at a tertiary cancer center. Key descriptive outcomes were: (1) Stakeholder perceptions: surveys (*n* = 17 clinicians) and focus groups (*n* = 5 patients, 2 carers) revealed strong perceived need and acceptability for routine screening. (2) Implementation metrics: 98% of approached patients (92/94) consented to screening. The baseline ePROM completion rate was 64.1% (59/92). PREMs from a subset (*n* = 9) indicated the tool was easy to use, though technical barriers (link expiration, missed notifications) were noted. (3) Clinical yield: Of the 59 respondents, 22.0% (*n* = 13) screened positive (PHQ‐2 ≥ 3) for depressive symptoms and 20.3% (*n* = 12) for at least mild anxiety (GAD‐7 ≥ 5). Through the stepped‐care model, 13 of 19 eligible patients accepted an intervention.

### 4.2 Interpretation

In this study, 22% of patients screened positive for depressive or anxiety symptoms, yet only 5% had a documented history of anxiety, and none had documented history of depression. This discrepancy suggests a limitation of opportunistic case finding, which relies on patients volunteering distress or clinicians recognizing it during time‐pressured consultations. In the context of EPCCTs, where patients with advanced cancer face poor prognoses and limited treatment options, denial and avoidance may serve as psychological protective mechanisms [21]. While systematic screening offers a complementary approach by proactively identifying individuals who might otherwise remain unnoticed, it is insufficient alone; as our data show, even when distress is identified, a third of patients declined support. This is consistent with literature reporting that a significant proportion of oncology patients who screen positive for distress decline help, often citing a preference to self‐manage or perceiving their distress as insufficiently severe [22]. This highlights that systematic screening may be a useful first step, but must be coupled with skilled, sensitive communication to explore patients' readiness to engage with support. The stepped‐care model, with initial low‐intensity CNS review, provides an opportunity for such exploration without mandating intervention, respecting patient autonomy while maintaining an open door to future support.

The high levels of stakeholder support align with clinical guidelines [7, 8] and suggest that psychological screening is considered a relevant component of care within the EPCCT context. The prevalence of psychological distress is consistent with prior research in similar cohorts [4, 10], indicating a reproducible area of unmet need that a structured pathway could potentially address.

An important finding for assessing feasibility is the baseline ePROM completion rate of 64%. This figure serves as a key implementation metric, indicating that while a functional workflow from invitation to intervention was established, a significant proportion of consented patients did not engage with the initial digital assessment. This rate is within the wide range of adherence (50%–100%) reported in ePROM implementation studies [23], reflecting a common challenge in digital health initiatives. The technical and logistical barriers identified are typical of early‐phase rollout and highlight specific, addressable targets for process optimization, such as streamlining invitation protocols and offering completion support.

The model demonstrated operability, as evidenced by the successful clinical review of all identified cases and the subsequent acceptance of support by 68% of those patients. This suggests the stepped‐care pathway is actionable within existing resources. However, the completion rate tempers conclusions about potential population‐wide impact, suggesting that feasibility is contingent not only on pathway design but also on strategies to support patient engagement.

The choice of a sensitive GAD‐7 cutoff of ≥ 5, while lower than some recommendations [19], was a deliberate decision aligned with our stepped‐care model. This approach, where a positive screen triggers a low‐intensity CNS consultation rather than an automatic specialist referral, allowed us to prioritize the identification of all potential cases while managing service resources effectively. This pragmatic strategy highlights the importance of aligning screening thresholds with the capacity and structure of the available supportive care pathway.

### 4.3 Considerations for Generalizability

This study was conducted at a single specialist center with an established ePROM infrastructure [15], therefore the results may be most relevant for similar research‐intensive settings. The EPCCT population itself is highly selected (advanced disease, prior treatment failure), which may influence both psychological distress profiles and engagement with supportive care. Therefore, while the identified barriers (resource concerns, technical issues) and the workflow model are likely transferable concepts, the specific acceptability and completion rates may vary in different healthcare environments or with less selected patient groups. Nevertheless, sharing implementation findings from specialist settings is important for the benefit of other institutions and clinicians working in comparable environments. By detailing a pragmatic approach to integrating psychological screening into EPCCT workflows, this study aims to contribute to shared learning rather than to assert effectiveness or prescribe a single model of care. This study provides a proof‐of‐concept and a methodological template for adaptation and evaluation in other centers.

### 4.4 Implications for Practice and Research

For practice, this study provides a replicable model for initiating screening in specialist EPCCT settings. To enhance feasibility, we recommend: (1) implementing targeted strategies to improve the ePROM completion rate, such as multimodal invitations (SMS/email), in‐clinic support for first completion, and proactive management of link expiration; (2) formally designating and training a role to oversee the screening workflow; and (3) integrating the stepped‐care algorithm into standard operating procedures to ensure consistency.

For research, the findings highlight several priorities. Firstly, multi‐center studies are needed to test the adaptation of this model and to obtain more robust estimates of engagement and distress prevalence across diverse settings. Secondly, future work should investigate the specific predictors of ePROM non‐completion in this population to inform engagement strategies. Finally, while this study focused on implementation outcomes, subsequent research should evaluate the effectiveness of such integrated screening pathways on longitudinal patient‐reported and clinical outcomes.

### 4.5 Limitations

In addition to the generalizability considerations noted, this study has limitations. The qualitative components may be subject to self‐selection and social‐desirability bias. The 64% completion rate introduces potential participation bias, meaning the reported prevalence of distress may not be fully representative of the entire cohort. The staff survey achieved a 63% response rate (17/27). While this is within expected ranges for voluntary surveys in clinical settings, the possibility of non‐response bias cannot be entirely excluded. However, the subsequent successful implementation of the screening pathway—with active engagement from the full multidisciplinary team in reviewing results and delivering stepped‐care interventions—provides pragmatic evidence of stakeholder acceptance that complements the survey findings. Furthermore, as a feasibility and implementation study, it was not designed or powered to establish causal effects on psychological outcomes.

## 5 Conclusion

This single‐center implementation study demonstrates that a routine ePROM‐based psychological screening pathway is operable and acceptable within the specialized context of EPCCTs. Key feasibility findings include strong stakeholder support, a functional identification‐to‐intervention workflow, and a baseline completion rate that identifies patient engagement as a critical focus for optimization. The model identified a proportion of patients with previously undocumented distress, supporting the potential value of systematic screening over reliance on opportunistic case finding in EPCCT populations. However, the 64% completion rate and the specialized setting necessitate a tempered assessment of broader feasibility. Future implementation efforts in similar contexts should couple this screening model with proactive strategies to support patient engagement and should be evaluated across multiple sites to better understand its generalizability and impact.

## Author Contributions

M.D.R. and R.Y. are joint first authors and have contributed equally. conceptualization: N.C., methodology: M.D.R., R.Y., N.C., A.S., formal analysis: M.D.R., R.Y., investigation: M.D.R., A.S., H.M., data curation: M.D.R., R.Y., A.S., H.M., L.C., D.C., M.K., F.T., writing – original draft preparation: M.D.R., writing – review and editing: M.D.R., R.Y., L.C., D.C., M.K., F.T., N.C., supervision: N.C., funding acquisition: N.C., All authors have read and agreed to the published version of the manuscript.

## Funding

The authors have nothing to report.

## Ethics Statement

The authors have nothing to report.

## Consent

Informed consent was obtained from all subjects involved in the study.

## Conflicts of Interest

The authors declare no conflicts of interest.

## Supporting information


Supporting Information S1
