# Supplementary material for: Psychological Screening and Support for Patients Enrolled in Early‐Phase Cancer Clinical Trials and the Possible Barriers: A Real‐World Tertiary Center Implementation Study
Source: Psychooncology. 2026 Apr 6;35(4):e70446. doi: 10.1002/pon.70446 (PMC13051752; doi:10.1002/pon.70446)
Supplement: Supplementary file 1 — Supporting Information S1 [file PON-35-e70446-s001.docx]

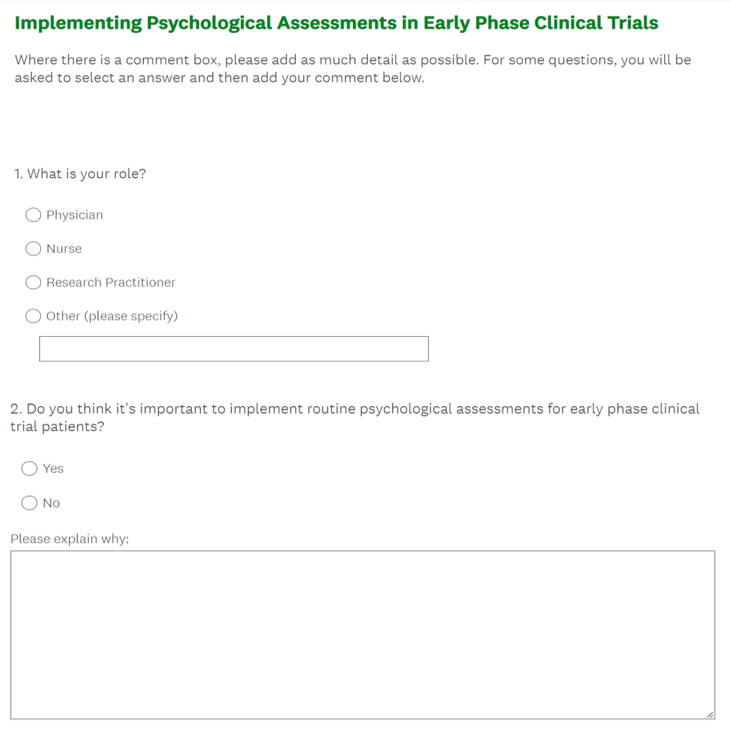

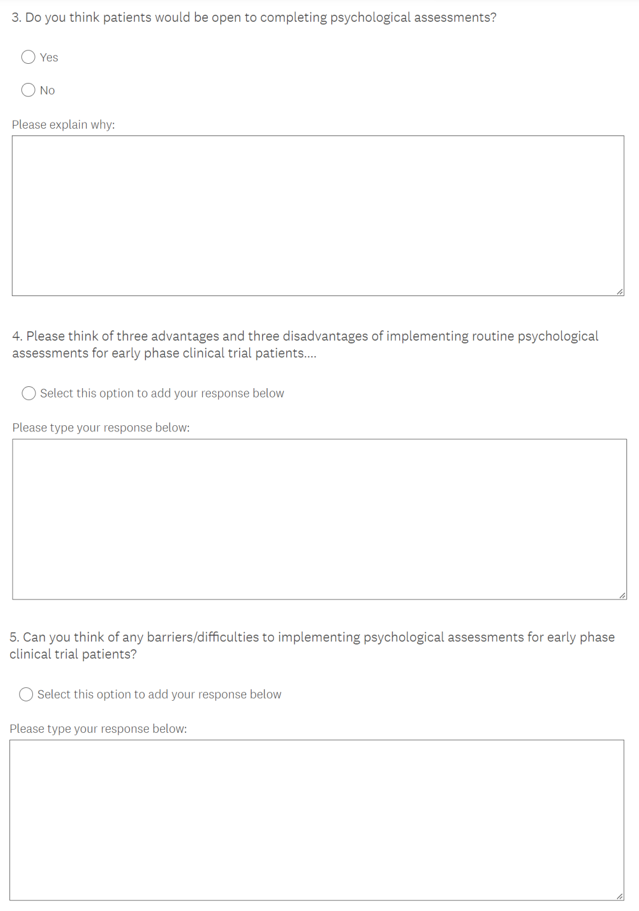

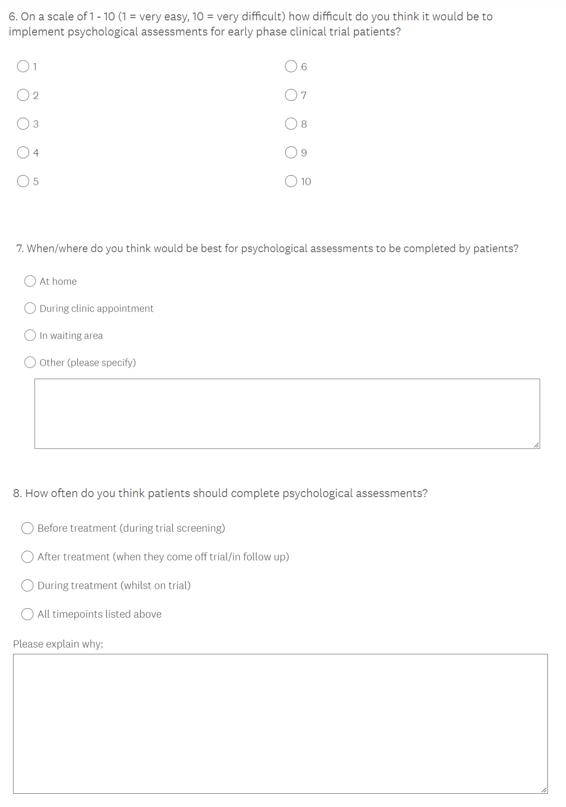

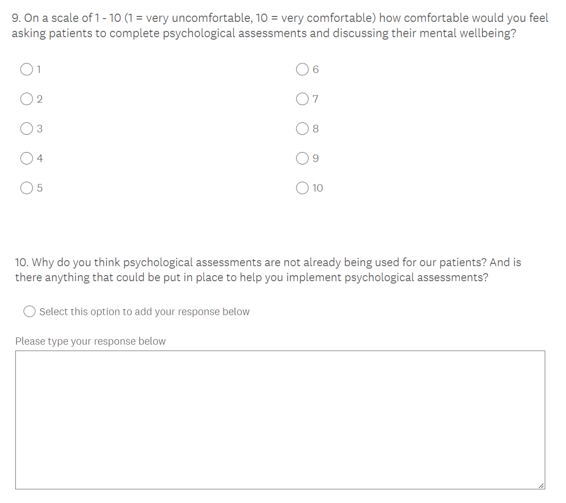


**Figure S1.** Staff questionnaire.


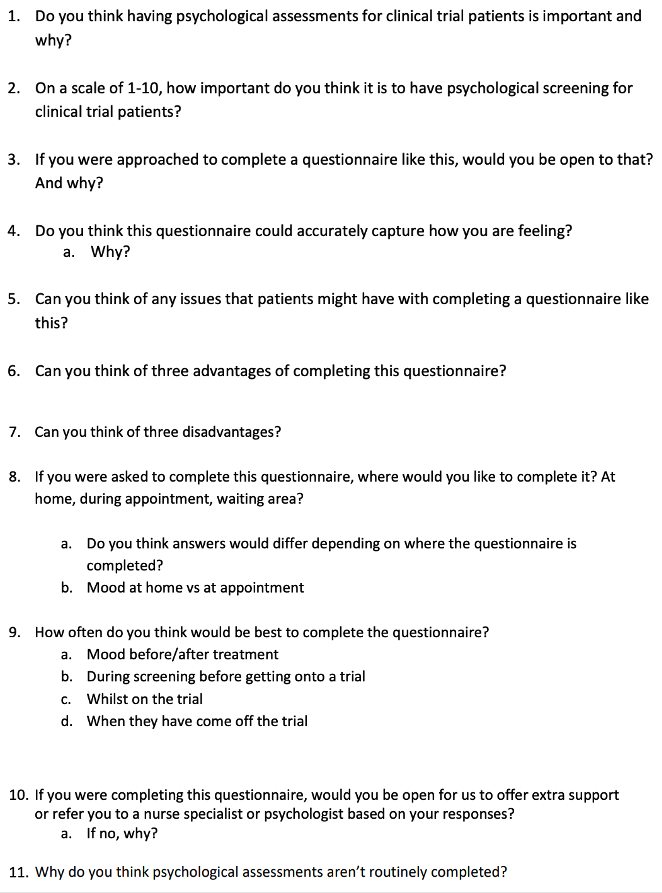


**Figure S2.** Patient and carer focus group questions


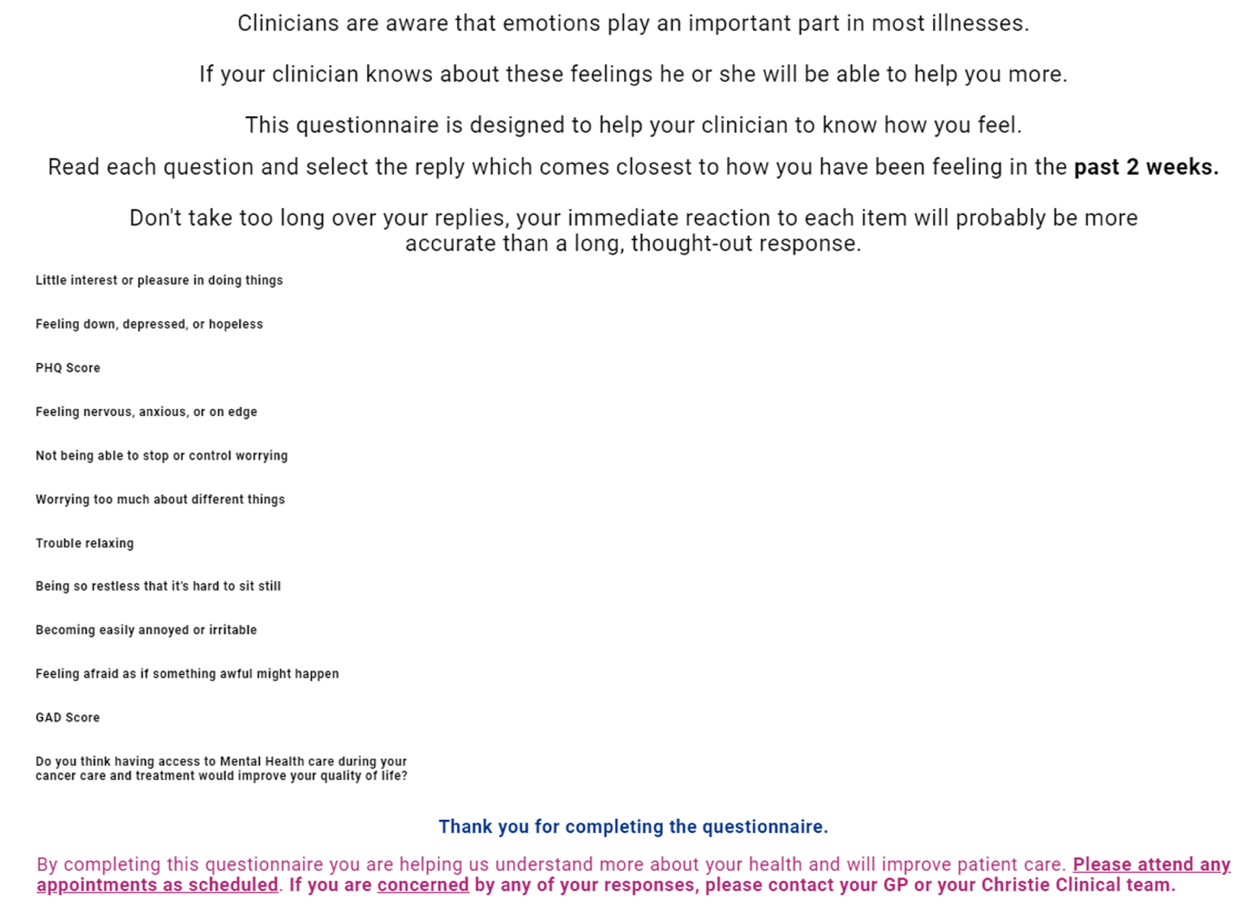


**Figure S3**. Psychological assessment ePROM questionnaire


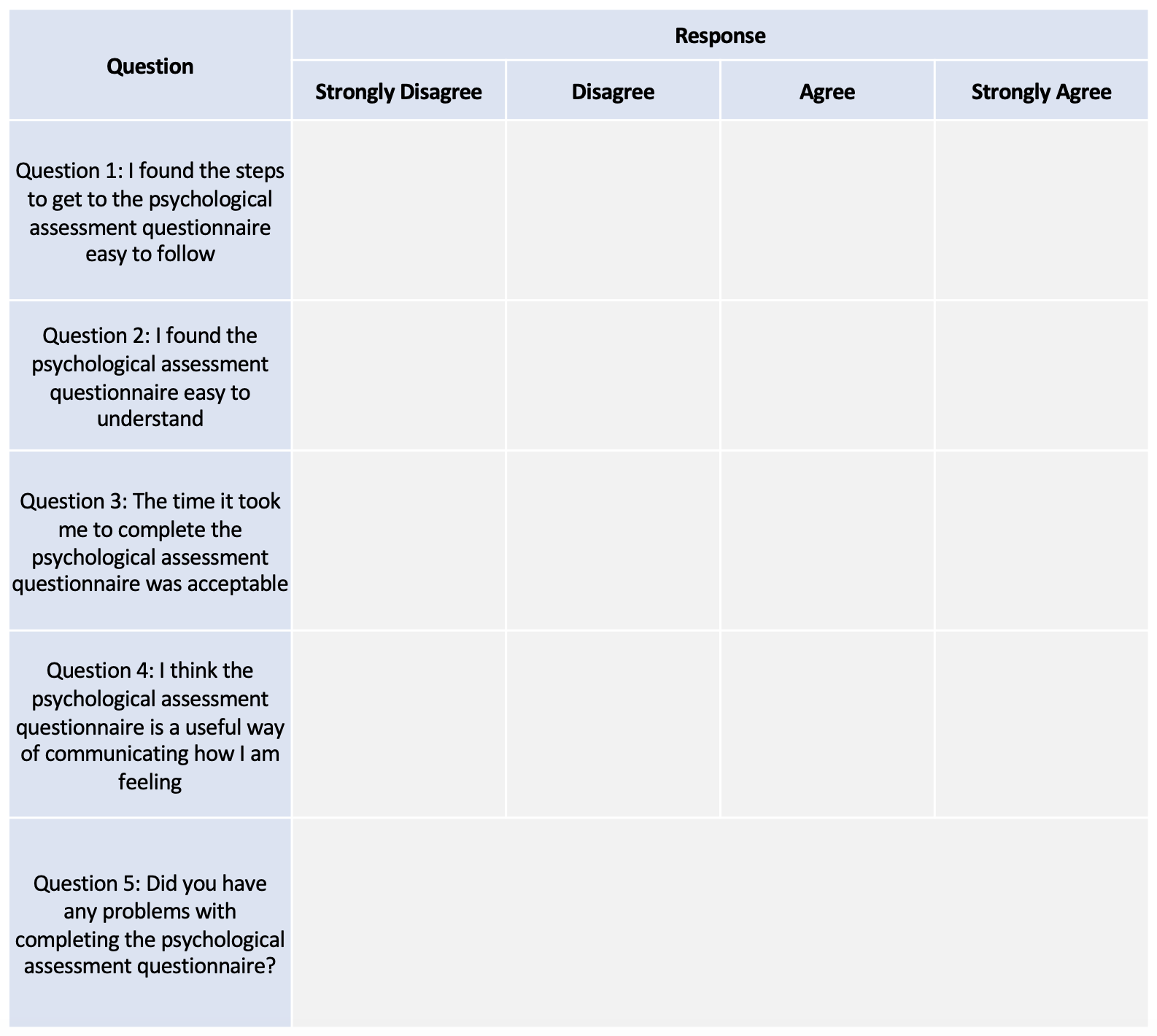


**Figure S4**. Patient-reported experience measure questionnaire


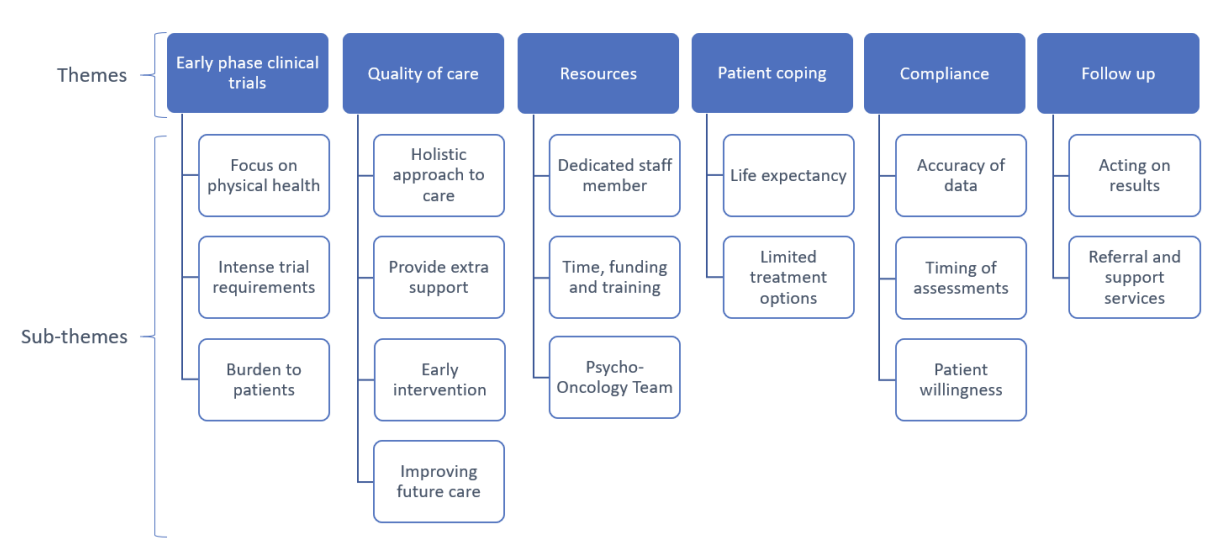


**Figure S5**. Primary themes and sub-themes of barriers to psychological assessments derived from a staff survey


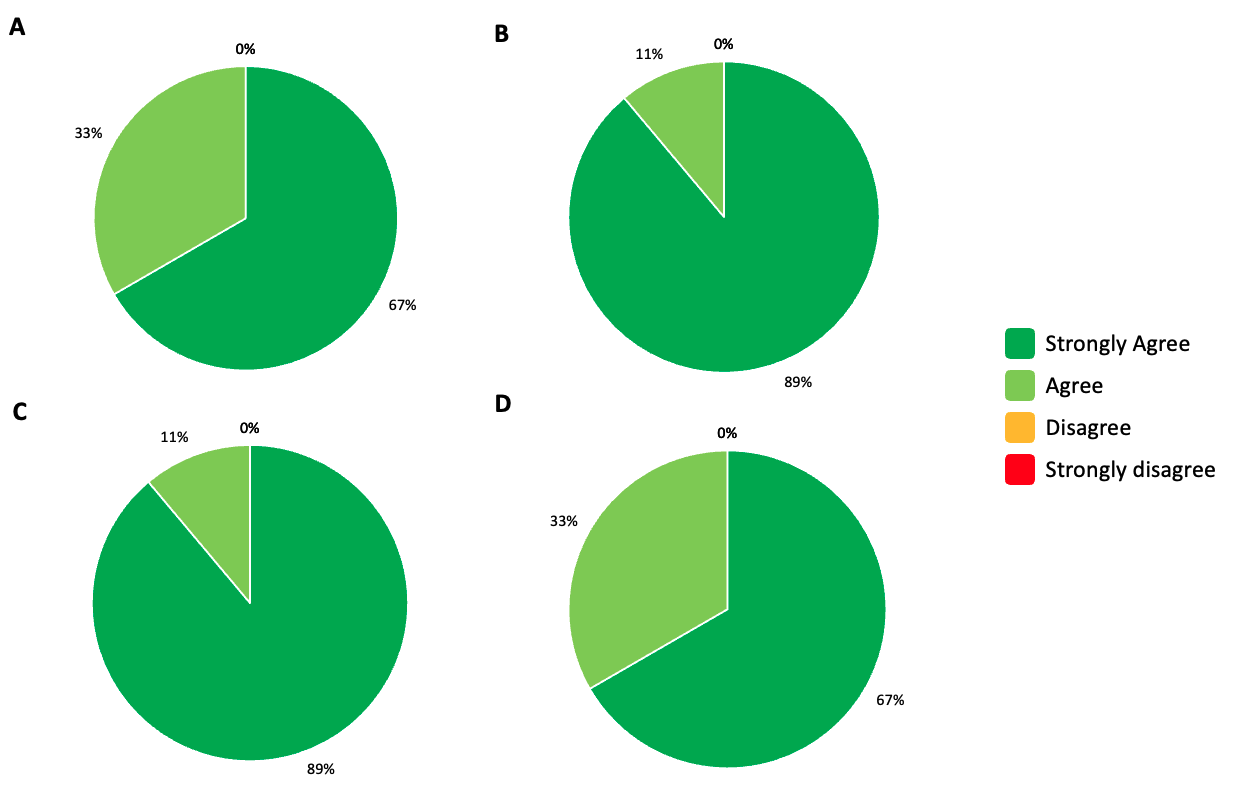


**Figure S6. Participant responses to a PREM questionnaire.** Each panel depicts the proportion of answers given to the following questions: (A) I found the steps to get to the psychological assessment questionnaire easy to follow; (B) I found the psychological assessment questionnaire easy to understand; (C) The time it took me to complete the psychological assessment questionnaire was acceptable; (D) I think the psychological assessment questionnaire is a useful way of communicating how I am feeling.
